# Prevalence and predictors of Pap smear cervical epithelial cell abnormality among HIV-positive and negative women attending gynecological examination in cervical cancer screening center at Debre Markos referral hospital, East Gojjam, Northwest Ethiopia

**DOI:** 10.1186/s12907-015-0016-2

**Published:** 2015-09-23

**Authors:** Melkamu Getinet, Baye Gelaw, Abinet Sisay, Eiman A. Mahmoud, Abate Assefa

**Affiliations:** Debre Markos Referral Hospital, Debre Markos, Ethiopia; Department of Medical Microbiology, School of Biomedical and Laboratory Sciences, College of Medicine and Health Sciences, University of Gondar, Gondar, Ethiopia; Department of Basic Sciences, College of Osteopathic Medicine, Touro University, Vallejo, CA USA

## Abstract

**Background:**

Cervical cancer is the leading cause of cancer related death among women in developing countries. Cervical cancer is preceded by cervical surface epithelial cell abnormalities (ECA) which can be detected by Pap smear test. Simultaneous human papillomavirus and human immunodeficiency virus (HIV) infection increases cervical cancer. Data on the prevalence and predictors of ECA among women in Ethiopia is limited. Hence, we aimed to determine the prevalence and associated factors of ECA among women.

**Methods:**

A comparative cross-sectional study was conducted among HIV+ and HIV- women attending gynecological examination in cervical cancer screening center at the Debre Markos referral hospital. The study subjects were stratified by HIV status and systematic random sampling method was used to recruit study participants. Cervical smears were collected for Pap smear examination. Logistic regression analysis was employed to examine the possible risk factors of cervical ECA.

**Results:**

A total of 197 HIV+ and 194 HIV- women were enrolled in the study. The overall prevalence of cervical ECA was 14.1 % of which the prevalence of atypical squamous cells undetermined significance (ASCUS), low grade squamous intraepithelial lesion (SIL), high grade SIL, squamous cell carcinoma and ASC, cannot exclude high grade SIL (ASCH) were 5.1, 3.8, 4.1 and 1.0 %, 0.0 % respectively. Significantly higher prevalence of ECA (17.8 %) was observed among HIV+ women (COR 1.9, 95 % CI: 1.1 − 3.4, *p* = 0.036) as compared to HIV-women (10.3 %). Multiple sexual partnership (AOR 3.2, 95 % CI: 1.1 − 10.0, *p* = 0.04), early ages of first sexual contact (<15 years) (AOR 5.2, 95 % CI: 1.5 − 17.9, *p* = 0.009), parity greater than three (AOR 10.9, 95 % CI: 4.2 − 16.8, *p* < 0.001) and long term oral contraceptive pills (OCP) use (AOR 11.9, 95 % CI: 2.1 − 16.7, *p* = 0.02) were significant predictors of prevalence of ECA.

**Conclusions:**

Cervical ECA is a major problem among HIV-infected women. Lower CD4+ T-cell counts of below 350 cells/μl, HIV infection, multiple sexual partnership, early age at first sexual contact, parity greater than three and long term OCP use were significant predictors of prevalence of ECA. Strengthening screening program in HIV+ women should be considered.

## Background

Squamous intraepithelial lesions (SIL) are an abnormal growth of squamous epithelial cells of the ecto-cervix. Cervical epithelial cell abnormalities (ECA) represent a spectrum of SIL that lie along the pathway, from mild-to-severe dysplasia to invasive cancer [1]. Cervical carcinoma develops gradually through well characterized precursor lesions [2]. Greater than 99.7 % cervical cancer is attributed by *human papillomavirus* (HPV) infection. HPV usually causes a variety of benign papillomatous lesions of the skin and mucosal basal epithelium [3, 4]. There are more than100 different HPV genotypes [5]. Based on oncogenic potential, HPV is classified as high-risk (HR) and low-risk (LR) oncogenic types. HR-HPV types, HPV 16, 18, 31, 33, 35, 39, 45, 51, 52, 56, 58, 59, 68 and 82, cause anogenital cancer [6], while infection with LR-HPV types, HPV 6 and 11, is associated with benign genital warts. HR-HPV types are detected in 99 % of cervical cancer, and about 70 % of cervical cancer is due to HPV 16 and 18 [7].

More than half of sexually active people become infected with HPV during their lifetime [8]. It is estimated that in Ethiopia about 33.6 % of women in the general population has HPV infection [9]. Persistent infection with HR-HPV types over time leads to the development and progression of cervical intraepithelial neoplasia (CIN). Not all women who acquire HPV infection do develop CIN. Rather approximately 90 % of HPV infections clear within 2 years [10]. The peak of HPV infection in women occurs in the late teens and early twenties following sexual exposure [11, 12]. Cervical cancer associated with HPV infection also leads to infertility. There is higher incidence of ECA among women complaining of infertility [13].

Cervical ECA can be detected and classified by cytological screening methods. Well organized programmes of regular gynecological screening and treatment of precancerous lesions have been very effective in preventing cervical cancer [14, 15]. Cytological examination of cervical scrapping from clinically suspicious cases by Papanicolaou (Pap) cytological screening test can detect cervical ECA. The Pap smear identifies any changes in cells of the transformation zone of the cervix [16]. The Bethesda System 2001 classifies ECA into atypical squamous cell (ASC), low-grade squamous intraepithelial lesion (LSIL), high-grade squamous intraepithelial lesion (HSIL) and squamous cell carcinoma (SCC). ASC comprises: ASC of undetermined significance (ASCUS) and ASC, cannot exclude HSIL (ASCH); LSIL encompasses: HPV, mild dysplasia, and CIN1; while HSIL includes: moderate and severe dysplasia, carcinoma in situ, CIN 2, and CIN 3. These categories promote specificity in the mode of treatment [17]. For patients with invasive lesions the stage of a cervical cancer is the most important factor in the selection of treatment modality. For women diagnosed with ASCUS and LSIL follow up with HPV-DNA testing, Pap smear or colposcopy within certain time interval is some of the management options. In general, noninvasive SIL identified using Pap smear only, are treated with superficial ablative procedures such as cryotherapy or laser therapy [18].

On a global level, 75 % of women has abnormal cervical cytology at least once in their life time which may progress to cervical cancer. Cervical cancer is the second most common women cancer worldwide of which 80 % occurs in developing countries. The higher prevalence of cervical ECA due to HPV was reported in African countries [19–21]. Current estimates indicates that in Ethiopia 4648 women are diagnosed annually with cervical cancer and 3235 die from the disease [9]. Several factors such as number of sexual partners and age of first sexual activity, smoking, immune-suppression, and presence of other sexually transmitted infection (STI) can increase the risk of developing cervical cancer [22]. Several studies revealed that human immunodeficiency virus (HIV) infection is associated with an increased risk of HPV related cervical ECA [23–25]. Mortality and morbidity due to cervical cancer is higher among HIV patients [26–28]. HIV infection and cervical cancer among women in Ethiopia are major public health problems. More than 534,000 adult women are estimated to be infected with HIV and at risk of developing cervical cancer. However, Ethiopia has invested little in the infrastructure, training, and laboratory capacity required for successful cytological screening [29]. Though studies have shown that the prevalence of ECA is more common among HIV-infected than non HIV-infected women, such data in Ethiopia is limited. For the development of a rational approach to the screening and the subsequent management of precancerous cervical lesion in HIV-infected women, understanding of the specific risk factors associated with ECA occurrence among HIV- positive women is very much important. The aim of this comparative study was to determine the prevalence of ECA and risk factors associated with its occurrence among HIV+ and HIV- women attending the Debre Markos referral hospital.

## Methods

### Study setting and design

A comparative cross-sectional study was conducted among HIV- and HIV + women attending at the Debre Markos referral hospital cervical cancer screening center from the 1^st of^ March to the 30^th^ of May, 2014. Debre Markos referral hospital is located in Debre Markos town in East Gojjam Zone 300 km North of Addis Ababa. According to July 2014 zonal statistical agency report, the town has an estimated population of 100,000. The hospital has cervical cancer screening and treatment center. The screening service is provided for all HIV+ women. All women attending the Debre Markos referral hospital during the study period for any gynecological problem were eligible for the study. Pregnant women, lactating women and women on menstrual cycle were excluded from the study. The sample size was determined using two population proportion formula with the assumption of 95 % confidence interval (CI), 5 % marginal error and 33.6 % prevalence of ECA among HIV-infected women (15) and 10 % nonresponse rate. Since there is no previous study done in Ethiopia among HIV- women, the prevalence of ECA was assumed to be 50 %. Using this assumptions the final sample size becomes 400 (200 HIV+ and 200 HIV- women). During the study period there were a total of 1600 HIV- and 2000 HIV+ women attending gynecological examination in cervical cancer screening center. The study subjects from both groups were selected by systematic random sampling method.

### Socio-demographic and clinical information

Data was collected after obtaining written informed consent from each participant. A pre-tested structured questionnaire was used to collect socio-demographic and clinical information needed for the study. Socio-demographic and clinical information included in this study were age, marital status, age at first sexual intercourse, number of sexual partners, duration of oral contraceptive pills (OCP) use, condom use, alcohol use, smoking, prostitution, history of STI, CD4 + T-cell count and parity.

### Cytopathological examination

The cervical smear specimens were collected by gynecologist. Cervical smears were taken with a wooden applicator stick, smeared on a microscopic slide, fixed immediately with 95 % ethanol and allowed to air dry. The smears were stained with Pap stain, examined and graded according to the criteria of Bethesda classification system [17]. To ensure the quality of Pap smear results, 20 randomly selected patients were evaluated by gynecologist with colposcopic examination and visual inspection. Moreover, representative smears were reexamined by pathologists at the University of Gondar hospital blinded from the first results. In this regard 30 randomly selected positive and negative slides were blindly rechecked by a pathologist.

### HIV test and CD4+ T cell count

HIV counseling and testing based the national guideline was offered to participants unaware of their HIV status. Whole blood sample of 8 ml was collected from each study subject for HIV testing and CD4+ T-cell counts. HIV testing was done based on current national rapid HIV testing algorithms. For all HIV+ women, CD4+ T-cell counts were determined by Fluorescent Activated Cell Sorter (FACS) count (Becton Dickinson) at Debre Markos referral hospital.

### Statistical analysis

Data was initially registered in a registration book and transferred to excel Microsoft spread sheet. Data was cleaned and checked for completeness before analysis using SPSS version 20 computer software. Descriptive statistical analysis was used to determine the socio − demographic and clinical characteristics of study participants and prevalence of cervical ECA. The prevalence of ECA was stratified by study subjects’ HIV status. Associations of patient characteristics with ECA were assessed using a series of bivariate logistic regression analysis. Then, to control simultaneously for the possible confounding effects of the different variables; a multivariable model was fitted with stepwise variable selection among variables having p-value ≤ 0.2 at bivariate analysis. In both bivariate and multivariate analyses, the associations were expressed in odds ratios (OR) and 95 % CI. For all cases p-value <0.05 were considered statistically significant.

### Ethical approval

The study was reviewed and approved by ethical review committee of School of Biomedical and Laboratory Sciences, College of Medicine and Health sciences, University of Gondar and official permission was obtained from Debre Markos referral hospital higher management. A written informed consent was obtained from each study participant. All Pap smear positive results were referred to the department of obstetrics and gynecology for immediate treatment. The patients record were made anonymous and any identifying information were removed prior to analysis. Individual records were coded and accessed only by research staff members.

## Results

### Consistency of microscopy and colposcopic examinations

Pap smear microscopic examination was undertaken initially by trained laboratory technologist. Results obtained were compared with results obtained by a pathologist and gynecologist for consistency. In this regard, ten positive Pap smeared slides were given to three of the readers and examined blindly. There was no discrepancy between the results of the laboratory technologist and the pathologist or the gynecologist results except for one sample that was diagnosed as LSIL by the laboratory technologist but as ASCUS by the pathologist (Table 1).

**Table 1:** Consistency of the results of the 3 readers

Sample No	Reader A			Reader B			Reader C		
	Method	Result	Grade	Method	Result	Grade	Method	Result	Grade
20	Msy	ECA	1	Msy	ECA	1	Cpy	ECA	-
55	Msy	ECA	2	Msy	ECA	2	Cpy	ECA	-
90	Msy	ECA	3	Msy	ECA	3	Cpy	ECA	-
111	Msy	ECA	1	Msy	ECA	1	Cpy	ECA	-
146	Msy	ECA	2	Msy	ECA	2	Cpy	ECA	-
244	Msy	ECA	2	Msy	ECA	1	Cpy	ECA	-
281	Msy	ECA	1	Msy	ECA	1	Cpy	ECA	-
307	Msy	ECA	1	Msy	ECA	1	Cpy	ECA	-
331	Msy	ECA	3	Msy	ECA	3	Cpy	ECA	-
343	Msy	ECA	1	Msy	ECA	1	Cpy	ECA	-

*Reader A* trained data collector, *Reader B* pathologist, *Reader C* Gynecologist, *Msy* Microscopy, *Cpy* Colposcopy, *ECA* Epithelial cell abnormality, *Grade 1* atypical squamous cell undetermined significance, *Grade 2* Low grade squamous intraepithelial lesion, *Grade 3* High grade squamous intraepithelial lesion, *Grade 4* squamous cell carcinoma

### Socio-demographic data and clinical characteristics of the patients

A total of 400 women (200 HIV+ and 200 HIV- women) were enrolled in the study, but 9 patients (3 HIV+ and 6 HIV-) were excluded because the smears were not adequate for evaluation. Therefore, further analyses were restricted to 391 study subjects. The mean age of the study subjects was 35.02 years with standard deviation of ±8.41 years. Two hundred and thirty (58.8 %) of the women were married, and only 65 (16.6 %) were employed. Majority of the study participants (59.1 %) were urban dwellers. Hundred and seventy six (45.0 %) of the women had no formal education (Table 2).

**Table 2:** Socio-demographic characteristics of women attending cervical cancer screening center at Debre Markos referral hospital

Characteristics	HIV status		Total n %	
	Positive n (%)	Negative n (%)		
Age( in year)				
<30	73(49.0)	76(51)	149	38.1
30-45	102(51.5)	96(48.5)	198	50.6
>45	22(50.0)	22(50.0)	44	11.3
Marital status				
Married	92(40.0)	138(60.0)	230	58.8
Single	10(32.3)	21(67.7)	31	7.9
Divorced	49(69.0)	22(31.0)	71	18.2
Widowed	46(78.0)	13(22.0)	59	15.1
Marital status				
Orthodox	185(52.4)	168(47.6)	353	90.3
Muslim	5(31.2)	11(68.8)	16	4.1
Protestant	7(31.8)	15(68.2)	22	5.6
Residence				
Rural	70(43.8)	90(56.2)	160	40.9
Urban	127(55.0)	104(45.0)	231	59.1
Educational status				
Illiterate	96(54.5)	81(45.5)	176	45.0
Primary school	59(58.4)	41(40.6)	101	25.8
Secondary& above	42(37.2)	71(62.8)	113	28.9
Occupation				
Employed	24(36.9)	41(63.1)	65	16.6
House wife/Farmer	81(41.9)	112(58.1)	193	49.4
No work	32(68.1)	15(31.9)	47	12.0
Daily laborer	20(90.9)	2(9.1)	22	5.6
Commercial sex worker	8(50.0)	8(50.0)	16	4.1
Others	32(66.7)	16(33.3)	48	12.3

*HIV* Human immunodeficiency virus

The number of life time sexual partners, greater than two, was higher in HIV+ women (66.6 %) as compared to HIV- ones (33.6 %). First sexual contact at early age (<18 years) was also higher among HIV+ women (56.0 %) than HIV- women (44.0 %). About 26.1 % of the study subjects had a history of STI. Two hundred and sixty seven participants (68 %) were accustomed to alcohol intake and 0.8 % was currently smoking. About 5.4 % of women used OCP for greater than 5 years and only 14.8 % of the women used condom. The number of parity greater than two was lower in HIV+ women compared to that of HIV- women (Table 3).

**Table 3:** Behavioral and clinical characteristics of women attending cervical cancer screening unit at Debre Markos referral hospital

Variable		HIV status		Total	
		Positive n (%)	Negative n (%)	N	%
No. of life time sexual partner	1-2	106(41.7)	148(58.3)	254	65.0
	>2	91(66.4)	46(33.6)	137	35.0
Age of 1^st^ sexual contact	<18	160(56.0)	126(44.0)	286	73.1
	18-20	25(32.0)	53(68.0)	78	19.9
	>20	12(44.4)	15(55.6)	27	6.9
Alcohol use	Yes	142(53.2)	125(46.8)	267	68.3
	No	55(44.4)	69(55.6)	124	31.7
Smoking	Yes	2(66.7)	1(33.3)	3	0.8
	No	195(50.3)	193(49.7)	388	99.2
History of STI	Yes	66(64.7)	36(35.3)	102	26.1
	No	131(45.3)	158(54.7)	289	73.9
Duration of OCP usage	<5 years	43(55.8)	34(44.2)	77	19.7
	>5 years	13(61.9)	8(38.1)	21	5.4
Condom use	Always	40(69.0)	18(31.0)	58	14.8
	Some times	52(46.8)	59(53.2)	111	28.4
	Never	105(47.3)	117(52.7)	222	56.8
Parity	≤2	122(52.6))	110(47.4)	232	59.3
	3-4	39(49.4)	40(50.6)	79	20.2
	>4	36(45.0)	44(55.0)	80	20.5

*HIV* Human immunodeficiency virus, *OCP* Oral contraceptive pills, *STI* Sexually transmitted infection

### Clinical examination and Pap smear results

Clinical investigation was also conducted for all study subjects and 16.4 % (64/391) were found to have abnormal clinical findings. Among patients who had abnormal clinical results, 60.9 %( 39/64) were positive for HIV. Abnormal vaginal discharge and contact bleeding were the most common clinical findings. In 56 (14.3 %) of the women abnormal vaginal discharge was observed, whereas 8 (2.0 %) of the women had contact bleeding. Pap smear examination revealed that 55 (14.1 %) patients were positive for cervical ECA. Higher prevalence of ECA (17.8 %) was observed in HIV+ women, of which the prevalence of ASCUS was 5.6 % (n = 11), 0.0 % ASCH, 6.1 % LSIL (n = 12), 5.1 % HSIL (n = 10) and 1.0 % SCC (n = 2). On the other hand, a 10.3 % cervical ECA prevalence was found among HIV- women with a prevalence of 4.6 % (n = 9), 0(0.0 %), 1.5 % (n = 3), 3.1 % (n = 6) and 1.0 % (n = 2) for ASCUS, ASCH, LSIL, HSIL and SCC respectively (Table 4).

**Table 4:** The prevalence of epithelial cell abnormality among women attending cervical cancer screening unit at Debre Markos referral hospital

Cervical cytology result		HIV status		Total tested	
		Positive	Negative	n	%
NIL		162(82.2 %)	174(89.7 %)	336	85.9
ECA		35(17.8 %)	20(10.3 %)	55	14.1
Types of ECA	ASCUS	11(5.6 %)	9(4.6 %)	20	5.1
	LSIL	12(6.1 %)	3(1.5 %)	15	3.8
	HSIL	10(5.1 %)	6(3.1 %)	16	4.1
	SCC	2(1.0 %)	2(1.0 %)	4	1.1

*NIL* Negative for intraepithelial lesion, *ECA* Cervical epithelial cell abnormality, *ASCUS* atypical squamous cell undetermined significance, *LSIL* Low grade squamous intraepithelial lesion, *HSIL* High grade squamous intraepithelial lesion, *SCC* squamous cell carcinoma, *HIV* Human immunodeficiency virus

**Fig. 1:**
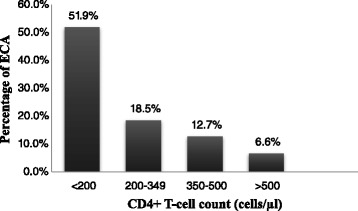
Proportion of cervical epithelial cell abnormality (ECA) compared with the CD4 + T-cell count level of HIV+ women. Increasing prevalence of cervical ECA was observed along with the concomitant decreasing of CD4 + T-cell count which indicates a direct relationship between the occurrence of cervical ECA and CD4 + T-cell count level

The prevalence of cervical ECA was high (51.9 %) among HIV+ women with CD4 + T-cell count <200 cells/μl. The prevalence of ECA among women with CD4 + T-cell counts of 200–349 cells/μl and 350–500 cells/μl were 18.5 % (n = 10) and 12.7 % (n = 7), respectively. On the other hand, relatively low prevalence (6.6 %) of ECA was found among women with CD4 + T-cell count greater than 500/ μl (Fig. 1).

### Risk factor analysis for cervical epithelial cell abnormality

Both bivariate and multivariate logistic regression analyses were employed to determine factors associated with ECA. All variables tested in the bivariate logistic regression analysis were entered into multivariate analyses if they have p-value of ≤0.2. The highest prevalence of cervical ECA (25.0 %) was observed in older age women (>45 years of old). Moreover, bivariate analysis showed that the prevalence of ECA was significantly higher among patients within the age groups of 30–45 years old and above (crude odds ratio (COR) 2.5, 95 % CI: 1.2 − 5.1, *p* = 0.012; COR 4.2, 95 % CI: 1.7 − 10.5, *p* = 0.002 respectively) as compared to younger women. Residence, educational status, condom use, smoking and alcohol consumption were not associated with the development of ECA.

The higher proportion of cervical ECA (63.6 %) was accounted by HIV+ women. Even though HIV infection was not found as an independent risk factor for ECA in multivariate analysis, in the bivariate analysis it was significantly associated with developing ECA (COR 1.9, 95 % CI:1.1 − 3.4, *P* = 0.036). A downward trend of the prevalence of ECA along the increment of CD4+ T-cell counts was observed among HIV+ women. Significantly higher prevalence of ECA were observed in HIV+ women with CD4+ T-cell counts <200 cells/μl (adjusted OR (AOR) 14.1, 95 % CI: 6.7 − 16.4, *p* < 0.001) and between 200 and 349 cells/μl all (AOR 9.6, 95 % CI: 1.8 − 11.5, *p* = 0.008) as compared to patients with CD4+ T-cell counts of above 500 cells/μl.

Women with a previous history of multiple lifetime sexual partners (more than two), were at high risk for developing ECA when compared to their counterparts with one or two sexual partner (AOR 3.2, 95 % CI: 1.0 − 10, *p* = 0.048). Early age at first sexual contact (<15 years) was also identified as a significant risk factor for the development of ECA (AOR 5.2, 95 % CI: 1.5− 17.9, *p* = 0.009). Association of marital status for the development of ECA was analyzed. Widowed (AOR3.2, 95 % CI: 1.2 − 8.8, *p* = 0.021) and divorced (AOR 3.0; 95 % CI: 1.1 − 8.1; *p* = 0.029) women were at higher risk than women who are married. Women with high parity (parity greater than four) were ten folds more likely to develop ECA (AOR10.9, 95 % CI; 4.2 − 16.8, *p* < 0.001) than women with parity lower than three. OCP users for more than five years were found to be at higher risk of developing ECA (AOR11.9, 95 % CI: 2.1 − 16.7, *p* = 0.02) (Table 5).

**Table 5:** Bivariate and multivariate analysis of risk factors for cervical ECA among women attending cervical cancer screening unit at Debre Markos referral hospital

Variables		Cervical ECA		COR (95 % CI)	P	AOR (95 % CI)	P
		Yes	No				
		No (%)	No (%)				
Age (in year)	<30	11(7.4)	138(92.6)	1		1	
	30-45	33(16.7)	165(83.3)	2.5(1.22, 5.11)	0.012	0.8(0.30, 2.09)	0.648
	>45	11(25.0)	33(75.0)	4.2(1.67, 10.47)	0.002	0.6(0.18, 2.25)	0.493
Marital status	Married	21(9.1)	209(90.9)	1		1	
	Single	1(3.2)	30(96.8)	0.3(0.04, 2.55)	0.290	1.2(0.11,12.43)	0.869
	Divorced	15(21.1)	56(78.9)	2.7(1.29, 5.50)	0.008	3.2(1.19, 8.77)	0.021
	Widowed	18(30.5)	41(69.5)	4.4(2.14, 8.91)	0.000	3.0(1.12, 8.09)	0.029
Education	NFE	35(19.8)	142(80.2)	2.6(0.87, 7.69)	0.087	2.2(0.69, 6.67)	0.181
	Primary	16(9.5)	152(90.5)	1		1	
Occupation	Employed	6(9.2)	59(90.8)	1		1	
	HW	28(18.3)	165(81.7)	1.8(0.29, 10.76)	0.275	0.4(0.10, 1.88)	0.536
	DL	10(16.7)	50(83.3)	2.9(0.64, 12.86)	0.167	0.3(0.05, 1.86)	0.205
	CSW	4(18.2)	18(81.8)	1.6(0.27,9.93)	0.004	0.1(0.01, 0.95)	0.046
	Others	7(14.6)	41(85.4)	2.6(0.80, 8.25)	0.081	0.8(0.17, 1.16)	0.112
Age of 1^st^ sex (in year)	<15	42(23.5)	137(76.5)	7.7(2.68, 22.28)	<0.001	5.2(1.49, 17.95)	0.009
	15-18	9(8.4)	98(91.6)	2.3(069, 7.77)	0.173	2.4(0.61, 9.49)	0.208
	>18	4(3.8)	101(96.2)	1		1	
No. of sexual partner	1-2	21(8.3)	233(91.7)	1		1	
	3-4	34(24.8)	103(75.2)	3.6(1.84, 6.96)	<0.001	3.2(1.00, 10.03)	0.048
History of STI	Yes	21(20.6)	81(79.4)	1.9(1.06, 3.53)	0.029	1.5(0.67, 3.42)	0.314
	No	34(11.8)	255(88.2)	1		1	
OCP user (in year)	<5	7(9.1)	70(90.9)	1		1	
	≥5	16(76.2)	5(23.8)	30.0(7.6, 118.37)	<0.001	11.9(2.11, 16.69)	0.020
	No	14(11.3)	110(88.7)	1		1	
Parity	≤2	13(5.6)	219(94.4)	1		1	
	3-4	6(10.7)	50(89.3)	3.2(1.36, 7.30)	0.007	1.7(0.53, 5.44)	0.362
	>4	36(35.0)	67(65.0)	7.9(3.67, 16.92)	<0.001	10.9(4.16, 16.75)	<0.001
HIV status	Negative	20(10.3)	174(89.7)	1		1	
	Positive	35(17.8)	162(82.2)	1.9(1.04, 3.38)	0.036	1.4(0.62, 3.16)	0.410
CD4 + T-cell count (cells/ μl)	<200	14(51.9)	13(48.1)	15.3(4.33, 54.31)	<0.001	14.1(6.69, 16.4)	<0.001
	200-349	10(18.5)	44(81.5)	3.2(0.95, 11.01)	0.060	9.6(1.79, 11.54)	0.008
	350-500	7(12.7)	48(87.3)	2.1(0.57, 7.52)	0.265	5.8(0.98,31.62)	0.052
	>500	4(6.6)	57(93.4)	1		1	

*HIV* Human immunodeficiency virus, *COR* crude odds ratio, *AOR* adjusted odds ratio, *CI* confidence interval, *P* p- value, *OCP* Oral contraceptive pills, *STI* Sexually transmitted infection, *NFE* No formal education, *CSW* Commercial sex worker, *HW* House wife, *DL* Daily laborer

## Discussion

The study showed that microscopic examination of Pap smear results by a trained laboratory technologist are comparable with the microscopic examination results of the same preparation observed by a pathologist. The current accepted practice is for the Pap smear to be examined by pathologist, while nurse responsibility is collection of the sample of cervical cells, and the technician responsibility is to prepare the slides with the pathologist responsible for slide readings and final reporting of findings. The comparable accuracy of the trained technologist reports of the Pap smear to the pathologist reports may indicate the possible utilization of trained technicians in the interpretations of Pap smears at the peripheral health facility where there is no pathologist.

In this study, 16.4 % of the women had abnormal clinical findings. The most prevalent clinical finding (13.4 %) was abnormal vaginal discharge but only 2.0 % of the women had contact bleeding. Vaginal discharge is often a normal and regular occurrence. There are, however, types of discharge that may suggest underlying infectious etiology. Such abnormal discharge was considered when the vaginal discharge was yellow or green in color, chunky in consistency, and have a foul odor. Most abnormal discharges in the study were caused by yeast or bacterial infection. The prevalence of abnormal gynecological findings such as abnormal vaginal discharge and contact bleeding of the current study was relatively lower than abnormal clinical findings reported from India which was 20 % and 6.7 % respectively [30].

In this study, the overall prevalence of cervical ECA based on Pap smear test was 14.1 % in which the prevalence of ASCUS, ASCH, LSIL, HSIL and SCC were 5.1, 0.0, 3.8, 4.1 and 1.0 % respectively. The prevalence of ECA among HIV+ women was 17.8 % which is quite higher than from that of ECA among HIV- women. Relatively concordant results on the prevalence of ECA among HIV-infected women were reported from Tanzania (17 %) [31] and Thailand (15.4 %) consisting of ASCUS 2.8 %, LSIL 8.5 %, and HSIL 3.5 % [32]. On the other hand, lower prevalence of ECA (2.8 %) was reported among Turkish women of which 2.2 % was ASCUS, 0.5 % LSIL, 0.1 % HSIL and 0.0 % SCC [33]. In different regions of Nigeria, Pap smear screening have shown a lower prevalence of HPV induced cervical ECA (7.6 − 13.2 %) [34, 35]. Similarly, based on Bethesda System ECA classification, the study conducted in Nigeria among young females indicated that the prevalence of ASCUS was 7 %, LSIL 12.2 %, HSIL 7.7 % and SCC 0.7 % [35]. Another study among Italian women also reported 2.8 % ASCUS, 6.2 % LSIL and 1.7 % HSIL cervical cytological abnormalities [36]. In contrast, higher prevalence of HPV induced cervical ECA was observed in South Africa in which 41.7, 70.2 and 83 % were ASCUS, LSILS and HSIL, respectively [21]. In another study carried among Turkish women, higher prevalence of ECA (54.8 %) was observed. The prevalence of ASCUS was 36.7 %, LSIL 16.8 %, HSIL 1.3 % [37]. The discrepancy in the prevalence of ECA between these studies may be due to differences in the study population. Higher prevalence of ECA observed from our finding and the report from South Africa may be due to the inclusion of high number of HIV-infected women. HIV is reported as one of the independent risk factor for development of cervical ECA and cervical cancer.

Women in the age groups of 30 years and older were at greater risk of developing ECA in the present study. There are also some other study findings which indicate that older age women had greater risk for the development of ECA. Cervical cancer mortality, usually occurring among unscreened women, increases with age, with the maximum mortality rate reported for white women between age 45 years and 70 years, and for black women in their 70s [38, 39]. Mortality among women with negative Pap smear screening is low at all ages. The prevalence of SCC and ASCUS were 36.4 and 81.8 % respectively among women <30 years of age. ASCUS was found to be highest in the youngest age group women in this study.

The study participants were stratified by their HIV status and data of 197 (50.4 %) HIV+ and 194 (49.6 %) HIV- women were analyzed. We found higher prevalence of cervical ECA (17.8 %) among HIV+ women as compared to HIV- women (10.3 %). This finding was very similar to the study finding reported from Brazil where ECA was more common in the HIV+ group (12.1 %) compared to the HIV- group (5.4 %) [40]. In a study finding reported from India, all cervical ECA (26.35 %) were found among HIV+ women [41]. HIV infection is one of the major risk factor that contributes for the growth of cervical ECA. HIV leads to an increased risk of CIN and cervical cancer [40]. Up to 20 % of HIV co-infected patients develop HPV-induced premalignant lesions of the uterine cervix within three years of HIV diagnosis [42]. Progression of an untreated HPV-induced dysplastic lesion can lead to invasive cervical cancer, an AIDS defining illness [43].

Moreover, the prevalence of ECA in this study was significantly higher among HIV+ women with lower CD4 + T-cell counts of < 350 cells/μl. Statistically significant downward trends of the prevalence of ECA along the increment of CD4+ T-cell counts in HIV+ women was observed in the present study. Similarly, higher prevalence of ECA among HIV+ women with lower CD + T-cell counts of <200 cells/μl was reported [40, 44]. There are study reports that documented the higher risk of cervical ECA when CD4+ T-cell counts fall below < 200 cells/μl [25, 28]. Decreased CD4+ T-cells count and increased HIV-RNA levels are risk factors for CIN. In addition, it has also been shown that with decreasing numbers of CD4+ T-cells, there is an increase in both frequency and severity of cervical dysplasia in HIV-infected women [36, 45]. Significant correlation was reported between low levels of CD4+ T-cells, high HIV-viral load and risk of CIN [46]. A Brazilian study demonstrated that immunosuppressed women had a higher risk of lesion recurrence as compared to women with a CD4+ T-cells count > 200 cells/μl [47].

In our study widowed (30.5 %) and divorced (21.1 %) women were significantly at higher risk for the development of ECA when compared to married (9.1 %) women. This difference might be due to divorced and widowed women may have multiple sexual partners when compared to married women. This finding is supported by the report from Ghana in which higher prevalence of ECA (21.3 %) was observed in polygamous women when compared to monogamous women (13.9 %) [48]. In this study, women with a history of STI were 1.5 times more likely to develop cervical ECA than women with no history of STI. Previous reports also demonstrated that genital infections were risk factors for the acquisition of HPV infection and the progression of cervical cancer [35, 43, 44].

We identified earlier initiation of first sexual contact (<15 years) as a significant risk factor for the development of ECA. Women with previous history of multiple life time sexual partners (more than two) were also at high risk for developing ECA which is supported by the study reported from Tanzania (44). Another most important finding of this study was that women with higher parity (greater than four) were 10.9 times more likely to develop ECA as compared to women with parity less than three. OCP users for more than 5 years had higher risk for the presence of ECA than their counterparts. Similar study supported that the prevalence of cervical cancer associated death in Ghana [48] among OCP users was higher.

### Limitation

The limitation of this study is that the number of study participants is relatively small for an epidemiological study; the results may only be applied to Northwest Ethiopia. Most of the study subjects were patients with gynecological problems which may not represent the general population. The result of this study also may not represent the hospital catchment population for most of women attending the cervical screening center are HIV+. The short duration of the study did not allow the adequate follow up of the disease progression. Moreover, the study didn’t further assess the etiology of the ECA.

## Conclusions

Women infected with HIV had a greater risk of developing cervical ECA than HIV- women. There was a downward trend of the prevalence of ECA along the increment of CD4+ T-cell counts among HIV-infected women. Lower CD4+ T-cell counts of below 350 cells/μl, earlier initiation of first sexual contact (at the age of <15 years), parity greater than four, being widowed and divorced, multiple sexual partnership (more than three partners) and long term OCP use were significant predictors of increased risk of cervical ECA. Hence, cytological screening program should be targeting specifically HIV+ women. Awareness creation on risk factors as multiple sexual partnership and sexual initiation at earlier age should be provided.

## Abbreviations

ASCUS: Atypical squamous cell undetermined significance
CIN: Cervical intraepithelial neoplasia
ECA: Cervical epithelial cell abnormality
HIV: Human immunodeficiency virus
HR: High risk
HPV: *Human papillomavirus*
HSIL: High grade squamous intraepithelial lesion
LR: Low risk
LSIL: Low grade squamous intraepithelial lesion
NIL: Negative for intraepithelial lesion
OCP: Oral contraceptive pills
SCC: Squamous cell carcinoma
SIL: Squamous intraepithelial lesion
STI: Sexually transmitted infection
